# 维奈克拉联合阿扎胞苷与地西他滨联合CAG方案治疗老年复发难治性急性髓系白血病的疗效及安全性比较

**DOI:** 10.3760/cma.j.cn121090-20250512-00224

**Published:** 2026-03

**Authors:** 占云 刘, 小鸽 楚, 光苹 董, 杰 郝, 秋生 陈, 赟翔 张

**Affiliations:** 1 上海市静安区北站医院血液科，上海 200070 Department of Hematology, Shanghai Jing'an District Beizhan Hospital, Shanghai 200070, China; 2 上海交通大学医学院附属瑞金医院血液科，上海市血液学研究所，上海 200025 Department of Hematology, Shanghai Institute of Hematology, Ruijin Hospital Affiliated to Shanghai Jiao Tong university School of Medicine, Shanghai 200025, China

**Keywords:** 维奈克拉, 抗肿瘤联合化疗方案, 白血病，髓系，急性, 复发难治, 老年, Venetoclax, Antitumor combination chemotherapy regimen, Leukemia, myeloid, acute, Relapsed/refractory, Elderly

## Abstract

**目的:**

比较维奈克拉（VEN）联合阿扎胞苷（AZA）（VA）与地西他滨联合CAG（D-CAG）方案治疗老年复发难治性急性髓系白血病（RR-AML）的疗效及安全性。

**方法:**

前瞻性随机研究，采用随机信封，计划入组50例。纳入2021年1月至2024年6月上海市静安区北站医院采用VA或D-CAG两种方案挽救治疗的老年RR-AML患者，主要终点为客观缓解率（ORR）、复合完全缓解（cCR）率，次要终点为总生存（OS）、持续缓解时间（DOR）和安全性分析。

**结果:**

50例老年RR-AML患者接受至少1个疗程VA或D-CAG方案治疗并完成疗效和安全性评估，VA组22例，中位年龄63.0（60.8～69.3）岁，D-CAG组28例，中位年龄66.5（62.3～69.0）岁。患者DNA甲基化基因突变频率最高，VA组阳性8例（36.4％），D-CAG组阳性9例（32.1％）。VA组和D-CAG组的1个疗程ORR分别为68.2％（15/22）和53.6％（15/28），cCR率分别为68.2％（15/22）和46.4％（13/28），两组患者1个疗程ORR和cCR率差异均无统计学意义（均*P*>0.05）。伴骨髓增生异常相关基因突变（AML-MR）患者中VA组cCR率为85.7％（6/7），高于D-CAG组的40.0％（4/10）。根据患者疾病状态分为早期复发、晚期复发及难治三组。结果显示，早期复发患者采用两种再诱导方案的cCR率均不到40％；而晚期复发患者VA组5例中4例获得cCR，D-CAG组2例均获cCR。中位随访时间为19（11～48.5）个月，整体患者中位OS时间为16个月。VA组和D-CAG组的中位OS时间分别为19个月和11个月，两组间OS差异无统计学意义（*P*＝0.189）。30例患者挽救化疗后达到cCR，VA和D-CAG组各15例，VA组患者DOR较D-CAG组存在显著优势（未达到对6个月，*P*＝0.023）。早期复发、晚期复发及难治三组的中位OS时间分别为5、21及18个月，晚期复发组患者疗效明显更具优势（*P*＝0.020）。4例挽救治疗后接受异基因造血干细胞移植患者均无病生存，与非移植患者相比预后存在优势（*P*＝0.007）。VA组皮疹（27.3％对3.6％，*P*＝0.047）及腹泻（40.9％对7.1％，*P*＝0.012）发生率均高于D-CAG组。

**结论:**

VA及D-CAG方案用于老年RR-AML的疗效相当，晚期复发的AML患者对于两种挽救化疗疗效优于早期复发及难治组。

## Abstract (en)

**Objective:**

To compare the efficacy and safety of venetoclax（VEN）combined with azacitidine（AZA）（VA）versus decitabine combined with cytarabine+aclarubicin+granulocyte colony-stimulating factor（CAG）（D-CAG）regimen in the treatment of elderly patients with relapsed or refractory acute myeloid leukemia（RR-AML）.

**Methods:**

This prospective randomized study was conducted using VA and D-CAG regimens for the salvage treatment of elderly patients with RR-AML. A sealed envelope was used for randomization, with a planned enrollment of 50 patients. The primary endpoints were objective response rate（ORR）and composite complete remission（cCR）rate, whereas the secondary endpoints included overall survival（OS）, duration of remission（DOR）, and safety.

**Results:**

From January 2021 to June 2024, 50 elderly patients with RR-AML received at least one cycle of either VA or D-CAG treatment and completed efficacy and safety assessments. The VA group comprised 22 patients with a median age of 63.0（range: 60.8–69.3）years, and the D-CAG group included 28 patients with a median age of 66.5（range: 62.3–69.0）years. DNA methylation gene mutations were the most frequent, comprising 8 cases（36.4％）in the VA group and 9 cases（32.1％）in the D-CAG group. The ORR was 68.2％（15/22）and 53.6％（15/28）and the cCR rate was 68.2％（15/22）and 46.4％（13/28）in the VA and D-CAG groups, respectively. No significant difference in ORR or cCR was observed between the two groups. Subgroup analysis revealed that the cCR rate in the VA group with AML-MR gene mutations was 6/7, which was higher than 4/10 in the D-CAG group. Patients were categorized into early relapse, late relapse, and refractory groups. The cCR rate for early relapse was below 40％ with both regimens. In the late relapse group, 4 of 5 patients in the VA group achieved cCR, and both patients in the D-CAG group achieved cCR. The median follow-up time was 19 months（*IQR*: 11–48.5 months）. The median OS was 16 months in the whole cohort, with 19 months for the VA group and 11 months for the D-CAG group（*P*＝0.189）. Among the 30 patients who achieved cCR after salvage chemotherapy（VA＝15 and D-CAG＝15）, the DOR in the VA group demonstrated a significant advantage over that in the D-CAG group（not reaching *vs* 6 months, *P*＝0.023）. The median OS for the early relapse, late relapse, and refractory groups was 5, 21, and 18 months, respectively, with significantly better efficacy observed in the late relapse group（*P*＝0.020）. Four patients who received salvage treatment followed by allogeneic stem cell transplantation had no evidence of disease, demonstrating a survival advantage compared with nontransplant patients（*P*＝0.007）. The incidence of rash（27.3％ *vs* 3.6％, *P*＝0.047）and diarrhea（40.9％ *vs* 7.1％, *P*＝0.012）in the VA group was significantly higher than that in the D-CAG group.

**Conclusion:**

The VA and D-CAG regimens have comparable efficacy for elderly patients with RR-AML. Patients with late relapse demonstrated better efficacy with both regimens compared with the early relapse and refractory groups.

急性髓系白血病（AML）是成人最常见的急性白血病类型，我国2022年统计显示其发病率约4.54/10万[1]，AML发病率会随年龄增加而升高，中位发病年龄为68岁[2]。老年患者由于机体状态和合并症问题，对传统高强度化疗耐受差，早期病死率高，和非老年AML相比，诱导化疗后不容易获得缓解，难治比例高，且更容易复发，整体预后差[3]–[4]，5年总生存（OS）率小于10％[2]–[3]。难治及复发一直是影响老年AML患者长期生存的最主要因素，如何选择治疗方案使老年患者获益更大始终没有定论。近年来低剂量地西他滨治疗老年AML有较多报道[5]，其联合CAG（D-CAG）等预激方案也被证实在复发难治性AML（RR-AML）中有一定疗效[6]。最新研究表明维奈克拉（Venetoclax，VEN）联合阿扎胞苷（Azacitidine，AZA）（VA方案）治疗RR-AML有较好的疗效及安全性[7]。本前瞻性研究旨在比较D-CAG与VA两种方案治疗不适合强化疗的RR-AML患者的疗效和安全性。

## 病例与方法

一、病例

2021年1月至2024年6月上海市静安区北站医院收治的50例老年RR-AML患者参与本研究，采用1∶1随机对照入组，随机方法为随机信封。主要入组标准包括：①年龄≥60岁；②参考WHO2022第5版AML诊断标准明确分型，均符合RR-AML诊断标准[8]；③以美国东部肿瘤协作组（ECOG）标准进行体能评分，所有患者入组前1周内ECOG评分0～3分。主要排除标准包括：①存在心肝肾严重器质性病变者；②合并除白血病外其他血液系统疾病、免疫系统等严重病变者；③存在化疗禁忌证、依从性差、严重精神疾患。以欧洲白血病网（ELN）2022标准[9]进行预后危险分层。本研究经上海市静安区北站医院伦理委员会批准（批件号：20210408001）。所有患者自愿参加本研究，本人或家属签署知情同意书。

二、分子生物学检测[10]

采用患者骨髓液2～4 ml，提取RNA和DNA。RNA用于检测RUNX1:RUNX1T1、CBFβ:MYH11、PML:RARα、BCR:ABL1、KMT2A重排、NUP98重排等；DNA用于检测AML相关基因突变，涵盖了KIT、FLT3、NPM1、CEBPA、DNMT3A、IDH1/2、TET2、TP53、RAS、JAK、AML-MR突变基因等。其中AML-MR突变指根据国际共识分类（ICC）-2022标准定义为“伴骨髓增生异常相关基因突变的AML”（AML-MR）所涉及的9个基因突变（ASXL1、BCOR、SF3B1、SRSF2、STAG2、EZH2、U2AF1、ZRSR2、RUNX1）。

三、治疗方案

1. 诱导治疗：VA方案：AZA 75 mg·m^−2^·d^−1^，第1～7天，皮下注射，同时口服VEN，100 mg第1天，200 mg第2天，400 mg第3～28天。为避免肿瘤溶解综合征，高白细胞水平的患者在开始治疗前需口服羟基脲，使WBC水平降至20×10^9^/L以下。D-CAG方案：地西他滨20 mg·m^−2^·d^−1^，第1～5天，静脉滴注；阿克拉霉素（Acla）14 mg·m^−2^·d^−1^，最大剂量20 mg/d，第3～6天，静脉滴注；注射用阿糖胞苷（Ara-C）15 mg/m^2^，每12 h 1次，第3～16天，皮下注射；重组人G-CSF 300 µg/d，第3～16天，皮下注射。化疗过程中若出现WBC>20×10^9^/L，则停用G-CSF，待WBC降低后恢复使用，不补充停药天数。

2. 巩固治疗：经过上述方案（第1疗程）获得完全缓解（CR）、CR伴血液学部分恢复（CRh）和CR伴血液学不完全恢复（CRi），继续原方案巩固治疗，每月1个疗程，直至复发，其中有条件移植患者建议尽早行异基因造血干细胞移植（allo-HSCT）；若获得部分缓解（PR），则重复原方案再次诱导，观察缓解情况；若NR，可交叉使用另一组方案重新诱导，或改用其他方案。对于疗效为形态学无白血病状态（MLFS）的患者，则暂缓治疗，直至患者血象恢复至中性粒细胞绝对计数（ANC）>0.5×10^9^/L且PLT>50×10^9^/L后，再次开始后续治疗。

3. 支持治疗：所有患者在治疗过程中予以止吐、护胃等对症处理，加强口腔、肛周护理。定期监测血常规、肝肾功能、电解质等。骨髓抑制期给予成分输血，若患者发生粒细胞缺乏感染，给予经验性抗生素治疗，对于有明确病原学依据的感染，根据药敏结果调整抗生素。

四、观察指标

治疗前行骨髓穿刺完善细胞形态学、免疫学、遗传学、分子生物学分型，化疗开始第28天复查骨髓涂片及微小残留病（MRD）评估疗效。治疗前后监测血常规、生化全套、心电图、心脏彩超、胸部CT。观察并记录患者出现的不良反应和用药后的血液系统及非血液系统不良反应。不良反应评估按照美国NCI-CTCAE 4.03标准执行。

五、疗效评定及随访

参照2022 ELN指南标准进行疗效评估。主要疗效评估终点为客观缓解率（ORR）、复合CR（cCR）率。ORR指疗效评判为CR、CRh、CRi、MLFS、PR的受试者比例。cCR率指疗效评判为CR、CRh、CRi的受试者比例。次要终点为总生存（OS）时间、持续缓解时间（DOR）和安全性分析。存活病例随访至2024年12月31日。随访方式包括电话随访、门诊复查、查阅病历，所有患者均未失访。OS时间指从治疗开始到任何原因死亡或末次随访时间。DOR指诱导治疗达CR到疾病进展或因任何原因死亡的时间。

六、统计学处理

采用SPSS 26.0统计学软件进行数据分析。计量资料用中位数（*IQR*）表示，数据符合正态分布，且方差齐，组间比较用独立样本*t*检验；不符合正态分布，组间比较用非参数检验。计数资料用（％）表示，采用卡方检验。生存分析采用Kaplan-Meier生存曲线及Log-rank检验。*P*<0.05为差异有统计学意义。

## 结果

1. 临床基线特征：50例老年RR-AML患者中，男26例、女24例。VA组22例，中位年龄63.0（60.8～69.3）岁，D-CAG组28例，中位年龄66.5（62.3～69.0）岁。两组患者的年龄、ECOG评分、ELN 2022预后分层、去甲基化药物（HMA）暴露、继发血液病、疾病状态、骨髓原始细胞比例、治疗前血象、基因突变数等差异均无统计学意义（均*P*>0.05）。D-CAG组患者既往接受的化疗疗程数大于VA组，且其在接受挽救化疗前PLT高于VA组。50例患者均检出驱动基因突变和（或）融合基因，检出基因突变中位数3（1～9）种，两组间差异无统计学意义（*P*＝0.654）。其中，DNA甲基化基因突变频率最高，DNMT3A突变共检出17例（34.0％），VA组8例（36.4％），D-CAG组9例（32.1％），两组间差异无统计学意义（*P*＝0.754）。其余高频基因突变包括IDH1/2 15例（30.0％），NPM1 15例（30.0％），FLT3-ITD 9例（18.0％），AML-MR突变17例（34.0％）。上述基因突变在两组间分布差异均无统计学意义（表1）。

**表1:** 50例老年复发难治性急性髓系白血病患者临床特征

临床特征	VA组 （22例）	D-CAG组 （28例）	*P*值
年龄［岁，*M*（*IQR*）］	63.0（60.8～69.3）	66.5（62.3～69.0）	0.259
性别（例，男/女）	9/13	17/11	0.164
ECOG评分［例（％）］			0.526
0～1分	3（13.6）	4（14.3）	
2分	12（54.6）	11（39.3）	
3分	7（31.8）	13（46.4）	
HMA暴露［例（％）］	14（63.6）	14（50.0）	0.335
继发血液病［例（％）］	1（4.5）	5（17.9）	0.211
疾病状态［例（％）］			0.248
早期复发	8（36.4）	10（35.7）	
晚期复发	5（22.7）	2（7.1）	
难治	9（40.9）	16（57.2）	
既往治疗疗程数［个，*M*（*IQR*）］	3（2～6.3）	6（2.3～9）	0.026
骨髓原始细胞比例［％，*M*（*IQR*）］	24.5（8.5～44）	26.5（9.5～40）	0.961
治疗前血常规［×10^9^/L，*M*（*IQR*）］		
WBC	2.7（1.2～3.4）	2.8（1.9～5.4）	0.204
中性粒细胞绝对值	0.9（0.26～2.2）	0.9（0.5～2.4）	0.363
PLT	60.0（38.8～102.2）	107.5（71.3～158.0）	0.034
ELN 2022危险分层［例（％）］		0.644
良好	9（40.9）	8（28.6）	
中等	6（27.3）	10（35.7）	
不良	7（31.8）	10（35.7）	
融合基因［例（％）］			
RUNX1::RUNX1L1	1（4.5）	2（7.1）	1.000
CBFβ::MYH11	2（9.1）	2（7.1）	1.000
分子突变［例（％）］			
NPM1	8（36.4）	7（25.0）	0.384
FLT3-ITD	4（18.2）	5（17.9）	1.000
DNMT3A	8（36.4）	9（32.1）	0.754
CEBPA bZIP	2（9.1）	1（3.6）	0.576
IDH1	1（4.5）	2（7.1）	1.000
IDH2	7（31.8）	5（17.9）	0.251
TP53	1（4.5）	1（3.6）	1.000
AML-MR	7（31.8）	10（35.7）	0.773

**注** VA：维奈克拉联合阿扎胞苷；D-CAG：地西他滨+阿克拉霉素+阿糖胞苷+重组人G-CSF；ECOG：美国东部肿瘤协作组；HMA：去甲基化药物；ELN：欧洲白血病网；AML-MR突变指以国际共识分类（ICC）-2022标准定义为“伴骨髓增生异常相关基因突变的AML”（AML-MR）所涉及的9个基因突变（ASXL1、BCOR、SF3B1、SRSF2、STAG2、EZH2、U2AF1、ZRSR2、RUNX1）

2. 疗效分析：50例患者均完成1个疗程诱导化疗。VA组和D-CAG组的1个疗程ORR分别为68.2％（15/22）和53.6％（15/28），1个疗程cCR率分别为68.2％（15/22）和46.4％（13/28），2个疗程cCR率分别为68.2％（15/22）和53.6％（15/28），两组患者1个疗程ORR（*P*＝0.295）和cCR率（*P*＝0.124）差异均无统计学意义（表2）。伴DNMT3A突变患者cCR率为47.1％（8/17），ORR为52.9％（9/17），两个治疗组间差异无统计学意义。伴AML-MR基因突变患者中，VA组cCR率为85.7％（6/7），高于D-CAG组40.0％（4/10），但差异无统计学意义（*P*＝0.134）。伴IDH2突变的患者中，VA组7例，4例CR，2例CRi；D-CAG组5例，2例CR。伴RUNX1突变患者中VA组2例均CR，而D-CAG组5例患者均未达CR。

**表2:** 老年复发难治性急性髓系白血病患者两种方案疗效比较

疗效	VA组 （22例）	D-CAG组 （28例）	*P*值
ORR［例（％）］	15（68.2）	15（53.6）	0.295
cCR	15（68.2）	13（46.4）	0.124
CR MRD−	7（31.8）	8（28.6）	
CR	5（22.7）	5（17.9）	
CRi	3（13.6）	0（0）	
MLFS	0（0）	0（0）	1.000
PR	0（0）	2（7.1）	0.581
不同疾病类型的cCR情况^a^			
早期复发	3/8（37.5）	3/10（30）	1.000
晚期复发	4/5（80.0）	2/2（100）	1.000
难治	8/9（88.9）	8/16（50）	0.088

**注** VA：维奈克拉联合阿扎胞苷；D-CAG：地西他滨+阿克拉霉素+阿糖胞苷+重组人G-CSF；ORR：客观缓解率；cCR：复合完全缓解；CR：完全缓解；MRD：微小残留病；CRi：CR伴血液学不完全恢复；MLFS：形态学无白血病状态；PR：部分缓解；NR：未缓解。^a^cCR患者/总患者（％）

根据患者疾病状态分为早期复发、晚期复发及难治三组。各组两个方案的疗效详见表2。晚期复发的AML对于上述两项挽救化疗疗效优于早期复发及难治组。

3. 生存情况：截至末次随访，中位随访时间为19（范围：11～48.5）个月。所有患者治疗流程见图1。整体人群中位OS时间为16（95％ *CI*：9.9～22.1）个月（图2A）。VA组和D-CAG组的中位OS时间分别为19（95％ *CI*：14.1～23.9）个月和11（95％ *CI*：8.4～13.6）个月，两组间OS比较差异无统计学意义（*P*＝0.189）（图2B）。共30例患者在挽救化疗后达到cCR，每组各15例；VA组的DOR更长，两组中位DOR分别为未达到和6（95％ *CI*：4.2～7.8）个月（*P*＝0.023）（图2C及2D）。

**图1:**
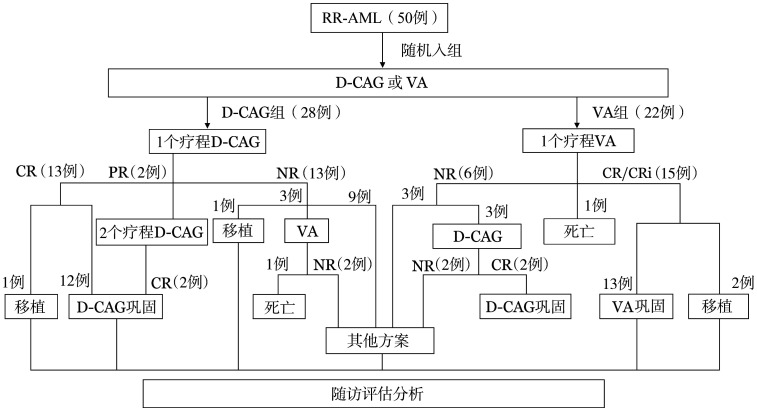
所有RR-AML入组患者治疗流程图 **注** RR-AML：复发难治性急性髓系白血病；VA：维奈克拉联合阿扎胞苷；D-CAG：地西他滨+阿克拉霉素+阿糖胞苷+重组人G-CSF；CR：完全缓解；CRi：CR伴血液学不完全恢复；PR：部分缓解；NR：未缓解

**图2:**
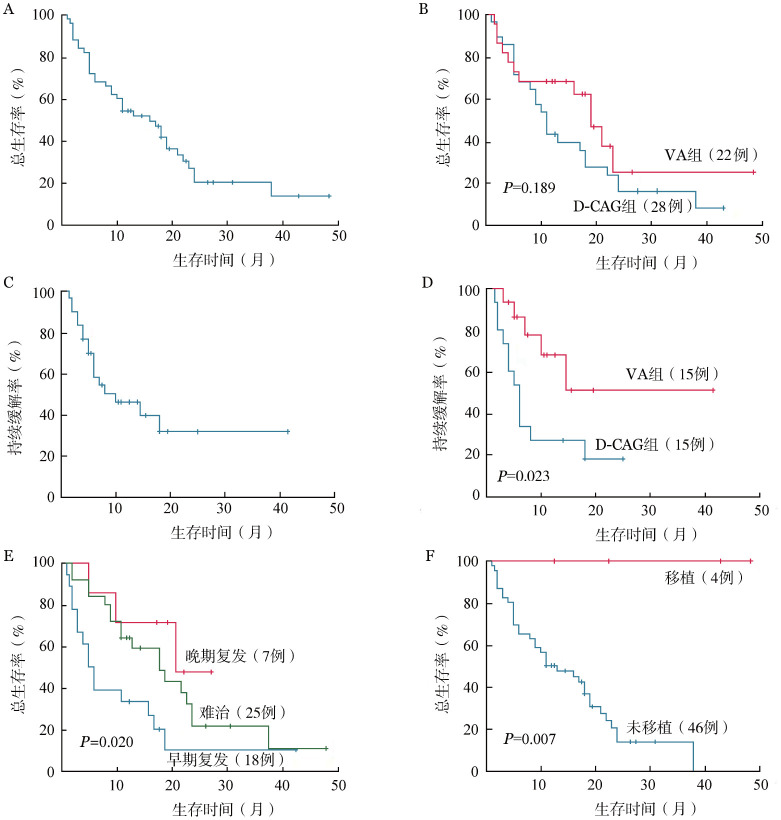
总人群及各亚组患者长期生存 **A** 所有患者的总生存曲线；**B** 不同治疗方案患者的总生存曲线；**C** 30例获得缓解患者的持续缓解时间；**D** 不同治疗方案患者的持续缓解时间；**E** 不同疾病状态患者的总生存曲线；**F** 移植组和非移植组患者的总生存曲线 **注** VA：维奈克拉联合阿扎胞苷；D-CAG：地西他滨+阿克拉霉素+阿糖胞苷+重组人G-CSF

根据患者疾病状态分为早期复发、晚期复发及难治三组。结果发现，三组的中位OS时间分别为5（95％ *CI*：2.9～7.1）个月、21个月及18（95％ *CI*：10.2～25.8）个月，晚期复发组患者OS明显存在优势（早期复发对晚期复发，*P*＝0.022；晚期复发对难治，*P*＝0.027）（图2E）。4例挽救治疗后桥接allo-HSCT患者均无病存活，与非移植患者相比预后存在显著优势（*P*＝0.007）（图2F）。

4.不良反应：

（1）血液学不良反应：VA组和D-CAG组中性粒细胞缺乏的持续时间（ANC低于0.5×10^9^/L恢复至任意2次血常规提示ANC>0.5×10^9^/L，以前次时间为准）分别为17（12～25.3）d和11（6.5～13.5）d，组间差异有统计学意义（*P*＝0.002）；PLT持续<20×10^9^/L时间（PLT<20×10^9^/L恢复至不输血前提下连续2次血常规提示PLT>20×10^9^/L，以前次时间为准）两组分别为0（0～7.8）d和24（20.5～30）d，组间差异有统计学意义（*P*＝0.002）。VA组患者在骨髓抑制期输注血小板数量更少，组间差异有统计学意义（*P*＝0.010）。

（2）非血液学不良反应：VA组皮疹发生率27.3％，高于D-CAG组的3.6％，差异有统计学意义（*P*＝0.047）；VA组腹泻发生率40.9％，高于D-CAG组的7.1％，差异有统计学意义（*P*＝0.012）。其他不良反应如恶心呕吐、便秘、腹痛、低钾血症及发热、咳嗽等的发生率差异均无统计学意义。此外，两组均出现Ⅲ级非血液学不良反应，VA组出现2例Ⅲ级肺部感染，1例Ⅲ级肝功能异常；D-CAG组出现1例咯血，1例尿路出血，1例肝功能异常，1例低钾血症。所有患者均未发生Ⅳ级非血液学不良反应。

## 讨论

老年RR-AML患者往往无法耐受高强度的再诱导化疗[11]–[14]，且多数后续移植意愿不大或不适合移植，故此人群整体预后差，中位生存期仅3～6个月。基于老年患者基础疾病多、合并用药复杂、脏器功能脆弱等特点，临床挽救方案应满足不良反应相对弱、药物相互作用少、不加重基础疾病的特点。目前指南推荐的不适合强化疗人群的挽救方案，除了针对特定基因突变的新型靶向药物外，仍以传统减低剂量化疗如单药AZA、小剂量Ara-C等为主[15]。而去甲基化药物联合预激方案或BCL-2抑制剂也逐渐被临床应用到RR-AML中。本研究旨在对比中国基层医院老年RR-AML患者使用VA和D-CAG两种方案的安全性和有效性。

地西他滨为DNA甲基转移酶抑制剂的一种，在骨髓增生异常综合征中的疗效首先得到认可，随后进一步证实了其在AML中的疗效[16]。Acla作为蒽环类抗生素，一直在AML的治疗中占据重要地位。Qiao等[17]最新研究证实Acla具有独特的抑制拓扑异构酶Ⅱ的机制，心脏毒性较低，并且Acla在柔红霉素或伊达比星耐药的AML病例中显示出较好疗效。既往报道RR-AML使用D-CAG方案挽救治疗ORR在50％～70％[18]–[19]。VEN作为抗凋亡蛋白BCL-2的抑制剂，能直接刺激肿瘤细胞线粒体凋亡，其在AML中的疗效亦得到关注。研究报道VA方案治疗RR-AML的ORR达55％～85％[20]–[21]。上述两种方案均具有较好的临床疗效和安全性，但在真实世界中针对不同RR-AML类型，或特定基因突变选择何种方案更受益，尚无明确报道。本研究分析对比发现，VA和D-CAG方案的疗效与既往临床研究结果相似，两种方案的整体疗效相当。

本研究中，3例VA方案再诱导无效的患者，采用D-CAG方案挽救治疗后有2例获得CR。本研究开展期间，6例VA方案初始治疗后复发患者接受D-CAG方案挽救后有3例获得CR，提示在VA耐药老年AML中，D-CAG可能为一种切实有效的挽救方案。VEN耐药可能与MCL-1上调或表观遗传异常相关，地西他滨通过去甲基化恢复抑癌基因表达并下调MCL-1，与AZA虽同为去甲基化药物，但与CAG方案联合应用仍可能会部分克服耐药。上述结果提示，对于RR-AML在方案选择上要尽量避免曾用过的药物，或者选择交叉组合用药，可能会提高疗效。对于D-CAG再诱导无效的3例患者，采用VA挽救治疗后1例因重度骨髓抑制感染死亡，2例无效。晚期复发患者对于上述两项挽救治疗，疗效均优于早期复发及难治组，这一结果强调了提高初治患者CR率及延长PFS尤为重要。

基于基因突变的亚组分析发现，AML-MR突变组接受VA方案治疗后CR率高于D-CAG组，可进一步扩大样本量验证。Matthews等[22]的一项回顾性研究分析了接受VA和CPX351治疗的656例AML患者，包括289例（56％）AML-MR患者，研究表明与CPX351方案比较，VA组能获得类似的OS。Lachowiez等[23]亦观察到此类患者采用VEN-HMA方案CR率达79％，明显高于传统化疗，该研究还特别提到SRSF2合并IDH2突变者生存获益更为显著。本研究中，IDH2突变患者接受VA方案治疗后DOR优于D-CAG组；其中有2例SRSF2合并IDH2突变患者，均在VA方案治疗后达CR，截至末次随访时均处于无病存活状态。Aldoss等[24]回顾分析了33例RR-AML老年患者使用VEN-HMA治疗的临床疗效，研究结果亦显示携带IDH1/2的患者治疗反应率较高，ORR达64％，1年OS率为53％。Lou等[25]报道了48例老年RR-AML患者采用VA方案治疗，研究结果显示治疗1个周期后cCR率为47.9％，所有患者中位OS时间达9.6个月。本研究结果与上述分析基本一致，均提示选择挽救方案时明确基因突变类型有较大价值。

VA组与D-CAG组的中位OS时间差异无统计学意义，但在挽救化疗后达到cCR的30例患者中，VA组DOR更长（*P*＝0.023）。4例患者接受allo-HSCT，在末次随访时都保持无病生存状态，与非移植患者相比有显著优势（*P*＝0.007），其中有1例60岁患者是NR状态下强行移植，亦取得了较好的疗效。Pollyea等[26]对比了VA方案治疗后获CR患者的生存获益，119例患者中有60例适合移植，其中21例接受了allo-HSCT，中位年龄65岁。与拒绝移植的患者相比，中位OS时间显著提升（未达到对588 d，*P*＝0.01）；与不适合移植组比较同样存在显著的中位OS优势（未达到对291 d，*P*<0.001）。上述发现强调了造血干细胞移植在RR-AML患者中改善长期预后的重要价值，即使对于老年患者，如体能状态改善、有移植条件时，仍建议通过移植改善长期预后。

两组最常见的不良反应为血液学和肺部感染，其次为消化道反应，均与既往报道一致。少数患者在治疗过程中出现严重而漫长的骨髓抑制，应根据患者的缓解及血象恢复情况，在后续疗程应适当缩短阿糖胞苷或维奈克拉用药天数。本研究观察到VA组皮疹、腹泻发生率较高，皮疹给予西替利嗪抗过敏对症处理后均好转，临床未予减量。腹泻均发生在服药第1～3天，口服蒙脱石散后均好转，后续服药耐受良好。以上提示我们需重视靶向药物应用后的不良反应并及时处理。

本研究提示，VA及D-CAG方案用于老年RR-AML的疗效相当，耐受性良好，但对于化疗后能够达到CR的患者，VA组可能有更优的无病生存。晚期复发的AML对于上述两种挽救化疗方案疗效优于早期复发及难治组。RR-AML患者的基因分型应作为后续治疗选择的重要依据，同时也要结合患者体能状况、经济条件、既往疾病史、药物暴露等因素。对于有VEN暴露史的患者，D-CAG可能是一个有效的挽救治疗方案。allo-HSCT仍然是老年RR-AML患者重要且可能治愈的治疗方式。
